# Progress in State Medicine

**Published:** 1899-02-25

**Authors:** 


### Progress in State Medicine.

### STATE MEDICINE.

**Disinfection.**—Much experimental work has been done during the past twelve months with a view to determining the value of formalin as a general disinfectant. Formalin is a 40 per cent. solution of formic aldehyde ($CH_2O$) a derivative of methylic alcohol. It may be used as a solution for the disinfection of surfaces in strengths of ½ or 1 per cent., or formalin vapour generated in specially constructed lamps from solutions, or from pastilles and tablets, may be employed for the disinfection of rooms. Harrington,[1] as the result of a series of elaborate researches, finds that formaldehyde vapour must be regarded and employed as a surface disinfectant only, and can never be anything else, in spite of its power of penetration under favourable conditions. A quart of formalin evaporated in a room of 2,250 cubic feet will destroy all exposed organisms within half an hour, and other organisms protected by cotton cloth coverings in one and a half hours. Through dry, pervious substances, such as cotton, hair, &c., the gas penetrates more or less easily, but in the presence of moisture its power of penetration is practically nil. Aronson,[2] experimenting with a lamp of his own design, arrived at very similar results. He used solid paraformaldehyde in the form of pastilles providing two grammes of the disinfectant for each cubic metre of air space. After exposure for twenty-four hours all the test objects in a room of 100 cubic metres were found to be sterilised. Objects in the centre of feather pillows were, however, not acted upon sufficiently, the penetrating power of the gas being limited. The poisonous effects of the gas on the higher animals is not marked, as rabbits left in the room during disinfection were not killed. Formalin vapour having about the same specific gravity as air does not tend to accumulate at any particular level of the room. It exerts no deleterious action on fabrics, wall papers, pictures, leather, or on instruments. Novy and Waite[3] pronounce formalin as superior to sulphur fumes, and give the following directions for its use for room disinfection: All openings should be tightly caulked; books, linen, blankets, &c., should be stretched on a line; walls and floors should be sprayed with water; five ounces of formalin should be distilled for each 1,000 cubic feet of space, and at least ten hours should elapse before the room is opened. In America the Kinyoun lamp is largely used, or some form of autoclave in which the aqueous solution, mixed with equal parts of 20 per cent. solution of calcium chloride, is boiled under a pressure of three atmospheres, the quantity of gas liberated into the room being regulated by the quantity of formalin added. Wright,[4] whilst acknowledging the efficiency of Trillat's autoclave, claims that the Alformant lamp is much simpler, cheaper, and more portable. He advises that 20 grammes of paraformaldehyde be used for every 1,000 cubic feet of space, and calculates the cost at threepence or sixpence per 1,000 cubic feet.

**Disinfection by Steam.**—Doty[5] describes some important modifications and improvements which he has made on steam disinfecting apparatus of Lyon's and Reck's type. The difficulty with such apparatus has been to obtain penetration of the superheated steam into the interior of packages with sufficient rapidity. As a rule, it takes 15 or 20 minutes to raise the temperature in the centre of a bulky body to 230 deg. Fahr., so that the exterior may be exposed for quite a lengthened period to a high temperature, and may undergo a certain amount of damage. To obviate this, and to accelerate penetration, a steam exhauster is connected with both the chamber and the jacket, and the first step in the process of disinfection consists in creating a vacuum of one and a half atmospheres, and this with Doty's arrangement can be done in one minute. The air having thus been exhausted from the chamber and its contents, steam is admitted, and the temperature rises in three or four minutes to 230 or 240 deg. Fahr. At the expiration of 15 minutes the steam exhauster is again used to secure the previous degree of vacuum. This removes the steam from chamber and contents, and then a fresh air inlet is opened, and a current of air drawn through for eight or 10 minutes, so that goods are taken out completely dried. The novel features about this process are (1) the creation of a vacuum; (2) the utilisation of a fresh air inlet. By the means of the former the time occupied in disinfecting is reduced by one-half, thorough penetration is ensured, and goods are but little affected. The utilisation of the fresh air inlet secures the thorough drying of materials so that clothing is immediately ready for use. The efficacy of the method was tested by registering thermometers in the interior of packages of paper, and by bacteriological cultures similarly protected. The results were in all cases eminently satisfactory. Delepine[6] points out the excellent results that may be obtained in disinfecting by current steam, *i.e.*, steam not under pressure. The apparatus he used consisted of a deep flat-bottomed dish filled with water and heated over a powerful Bunsen burner, whilst over the dish there was placed a metallic bell-jar with a conical top, into which was fitted a cork perforated so as to permit of the escape of steam, and to carry a thermometer. With such an apparatus better results were obtained than with an autoclave or with a Koch's steriliser. It is essential that a powerful burner be used so that the water boils quickly and the air is expelled. The walls of the steam chamber must be protected from cooling influences in order to reduce condensation to a constant quantity, and the outlet must be large enough to allow a free escape of steam. The temperature varied from 96 deg. to 99 deg. C., but the bactericidal action was invariably perfect provided care had been taken to expel all air from the chamber. Hyorth's "intense oil furnace" was used for heating the water in larger machines.

[1] Amer. Jour. Med. Sci., Jan. 1898. [2] Zeitschrift für Hygiene, xxv. p. 168. [3] The Medical News, May 21, 1898. [4] The Therapist, May 14, 1898. [5] Amer. Jour. of Med. Sci., Aug. [6] The Medical Chron., Feb. 1898.

